# "The Hospital," Elementary Schools and the Public

**Published:** 1886-10-16

**Authors:** Hugh P. Maxwell

**Affiliations:** Cromwell Road, Redhill


### "*THE HOSPITAL*," ELEMENTARY SCHOOLS AND THE PUBLIC.

THE appearance of THE HOSPITAL marks a new era in periodical literature. It is a most useful and helpful publication. Its introduction into the higher classes of elementary and other schools would produce very beneficial effects on the minds of the youth of both sexes.

The serial, written with an obvious purpose, cannot but excite compassion for suffering humanity. The notice of the late learned and urbane Editor of *The Lancet*, who never seemed to tire of doing good, must stimulate and encourage the readers thereof to go and do likewise, even in a lesser degree.

Undoubtedly, the intelligent classes of society have now unanimously decided that THE HOSPITAL supplies a distinct and long-felt want, and is sure to meet with the support it deserves. *Palmam qui meruit ferat.* HUGH P. MAXWELL.

Cromwell Road, Redhill.

